# Nasal septum morphology after surgically assisted rapid maxillary expansion with different expanders: A retrospective tomographic analysis

**DOI:** 10.1007/s10006-026-01652-3

**Published:** 2026-10-23

**Authors:** Júlia Ferreira Folhes, Christian Ruben Cusi Fernandez, Carlos Augusto de Jesus Oliveira Gonçalves, Mario Cappelette Júnior, Daniel Falbo Martins de Souza, Nicolas Homsi, José Thiers Carneiro

**Affiliations:** 1 Postgraduate Program in Dentistry, Department of Oral and Maxillofacial Surgery, Federal University of Pará, Belém, PA Brazil; 2 Department of Otorhinolaryngology – Head and Neck Surgery, Federal University of São Paulo, São Paulo, SP Brazil; 3 Oral and Maxillofacial Surgery and Traumatology Unit, Samaritano Hospital, São Paulo, SP Brazil; 4 Department of Surgical and Diagnostic Sciences, School of Dentistry, Marquette University, Milwaukee, WI USA

**Keywords:** Palatal Expansion Technique, Nasal Septum, Tomography

## Abstract

**Objectives:**

to evaluate, through tomographic analysis, whether surgically assisted rapid maxillary expansion (SARME) promotes changes in the degree of nasal septal deviation in adult patients, and to investigate whether these changes are influenced by the type of expander used.

**Materials and methods:**

this retrospective, observational, and quantitative study included 33 adult patients submitted to SARME and treated with either a Hyrax expander (*n* = 21) or a transpalatal distractor (*n* = 12). Computed tomography scans were analyzed at the preoperative period (T0) and six months postoperatively (T1). Nasal cavity height and nasal septum contour were measured, and their ratio was used to determine the degree of septal deviation. Measurement reliability was assessed using the intraclass correlation coefficient.

**Results:**

Inter-examiner reliability was excellent (ICC 0.938–0.974). No statistically significant differences were demonstrated in intragroup or intergroup analyses. From T0 to T1, nasal cavity height changed by +0.14 mm (Hyrax) and −0.01 mm (transpalatal distractor), septal contour by −0.34 mm and +0.14 mm, and NSD by −0.011 and +0.003. Intragroup p-values ranged from 0.070 to 0.982, and intergroup p-values at T1 were 0.932, 0.936 and 0.901.

**Conclusion:**

SARME does not promote significant changes in nasal septal deviation in adult patients, regardless of the type of expander used. Biomechanical differences between tooth-borne and bone-borne expanders do not appear to influence this outcome, indicating that SARME can be performed without concern for inducing or worsening septal deviation in adults with transverse maxillary deficiency, and that the choice of expander may be guided by other clinical criteria

**Clinical Relevance:**

these findings suggest that SARME can be safely performed without concern for inducing or worsening nasal septal deviation, supporting its use in adult patients with maxillary atresia, regardless of the expander type selected.

## Introduction

Transverse maxillary deficiency is a dentofacial deformity characterized by transverse narrowing of the maxillary dental arch, frequently resulting in unilateral or bilateral posterior crossbite, anterior dental crowding and a high-arched palate. This condition may compromise both facial aesthetics and respiratory function, owing to the associated reduction in nasal cavity dimensions [1].

Surgically assisted rapid maxillary expansion (SARME) is used to correct transverse maxillary deficiency in skeletally mature individuals, in whom the ossified maxillary sutures resist conventional orthodontic expansion. The procedure typically combines a Le Fort I–type osteotomy without downfracture of the maxilla with a median palatal osteotomy and the placement of an expansion device, and is recommended to achieve adequate outcomes in adult patients [2, 3].

The impact of SARME on craniofacial structures beyond the palate is well established in the literature. Transverse maxillary expansion results in extensive anatomical modifications, including an increase in the volume and width of the nasal cavity [4]. This anatomical remodelling raises questions regarding the behaviour of the nasal septum, a structure essential for the regulation of airflow and upper airway patency, and potentially susceptible to the forces dissipated during maxillary disjunction.

The nasal septum is an osteocartilaginous partition formed anteriorly by the quadrangular cartilage and posterosuperiorly by the vomer and the perpendicular plate of the ethmoid bone, resting inferiorly on the maxillary crest along the intermaxillary suture. Because the vomer articulates directly with the palatine processes of the maxilla, the septum lies within the load path of the forces generated during transverse expansion. As early as 1961, Haas reported that opening of the intermaxillary suture promotes separation of the nasal walls from the septum, accompanied by displacement of the nasal floor and lateral inclination of the alveolar process [5]. Although observational studies suggest that most adult patients exhibit only subtle changes in this structure, robust evidence confirming this behaviour in a consistent manner remains scarce [6].

The choice of the expansion appliance may influence the pattern of palatal expansion. Among the available devices, both tooth-borne appliances, such as the Haas and Hyrax models, and bone-borne appliances, known as transpalatal distractors, may be used [7]. Transpalatal distractors have been widely employed on the hypothesis that they promote greater skeletal expansion and reduce the complications commonly associated with tooth-borne devices, such as dental tipping, root resorption, cortical fenestration and orthodontic relapse [8].

Given the relevance of the nasal septum to upper airway patency, the effects of maxillary movement on this structure warrant investigation, particularly in adult patients. The distinguishing feature of the present study is the comparative analysis of the effects of tooth-borne and bone-borne expanders specifically on the nasal septum. Because different devices promote distinct patterns of skeletal expansion, it is important to determine whether these biomechanical differences influence the degree of septal deviation.

Accordingly, the aim of this study was to investigate, through a retrospective evaluation of tomographic examinations, whether the degree of nasal septal deviation changes after SARME and whether any such change is related to the type of orthopaedic expander used. The null hypothesis was that there is no significant difference in the change in nasal septal deviation between patients treated with a Hyrax expander and those treated with a transpalatal distractor.

## Methods

This is a retrospective, observational, quantitative clinical study with a hypothetical-deductive approach, conducted and reported in accordance with the Strengthening the Reporting of Observational Studies in Epidemiology (STROBE) guidelines for observational studies [9]. Tomographic examinations were collected from patients who underwent surgically assisted maxillary expansion between 2019 and 2022.

### Ethical aspects

This retrospective study was conducted within the scope of the Post-Graduate Program in Dentistry at the Federal University of Pará (UFPA) and was submitted for review and approval by the Institutional Research Ethics Committee in coparticipation with the Research Ethics Committee of Ophir Loyola Hospital, under approval number 8.058.393. All procedures followed the principles of the Declaration of Helsinki and complied with Brazilian National Health Council (CNS) Resolutions No. 466/2012 and No. 580/2018.

### Sample

The sample of this retrospective observational study was non-probabilistic and based on convenience, initially consisting of 35 adult patients (≥ 18 years) with transverse maxillary deficiency who underwent Surgically Assisted Rapid Maxillary Expansion (SARME). Due to the retrospective nature of the study, the potential for selection and information bias was acknowledged; however, this was minimized through strict inclusion criteria and standardized tomographic analysis.

Patients treated with either a transpalatal distractor or a Hyrax expander were included if they presented tomographic examinations in DICOM format obtained at two time points: T0 (preoperative) and T1 (6 months postoperatively). Additional inclusion criteria were the absence of previous surgeries or trauma involving the maxillofacial region, complete clinical records, and adequate image quality for analysis.

Patients with craniofacial syndromes or congenital anomalies, tomographic examinations with artifacts compromising image evaluation, or incomplete clinical data were excluded.

During the selection process, two patients were excluded from the initial sample, both belonging to the transpalatal distractor group, due to incomplete clinical information: these patients did not undergo the postoperative examination at the appropriate time. Thus, the final sample consisted of 33 patients, of whom 21 (63.64%) were treated with a Hyrax expander and 12 (36.36%) with a transpalatal distractor.

### Data collection

Clinical records of 33 patients with transverse maxillary deficiency greater than 7 mm, who underwent surgery between 2019 and 2022, were included in this retrospective study. All patients were treated with SARME performed by surgical teams led by two experienced surgeons. The surgical protocol was standardized and consisted of a bilateral Le Fort I osteotomy without pterygomaxillary disjunction, associated with an additional interapical osteotomy between the maxillary central incisors. A thin straight chisel was positioned between the incisors and directed palatally to promote rupture of the median palatal suture. No nasal septum release was performed. The only difference between treatments was the type of expander used.

The sample was divided into two groups according to the orthopedic device employed. Group 1 (G1) consisted of 12 patients treated with a transpalatal distractor (Rapid Palatal Expander, KLS Martin GmbH + Co. KG, Tuttlingen, Germany), while Group 2 (G2) included 21 patients treated with a Hyrax expander). After a latency period of seven days, activation of the expansion device was initiated. Patients in both groups were instructed and trained to perform activations of 1 mm per day. After achieving the required expansion, the appliances were locked and maintained in place for 6 months to allow bone consolidation.

Facial computed tomography (CT) scans were obtained using a multislice scanner (Aquilion 64, Toshiba America Medical Systems, Tustin, CA, USA), with exposure parameters of 120 kV and 132–140 mAs, and an acquisition time ranging from 10 to 15 s. The mean voxel size was 0.38 × 0.38 × 0.43 mm, with a slice thickness of 0.5 mm. All images were archived in Digital Imaging and Communications in Medicine (DICOM) format. CT examinations were performed at two distinct time points: T0 (preoperative) and T1 (six months postoperatively).

### Evaluation of nasal septal deviation

For quantitative analysis of nasal septal deviation (NSD), multislice CT scans were evaluated in the coronal plane using Weasis DICOM Viewer, version 4.5.1 (open-source software, https://weasis.org). Previously validated methodologies described in the literature were applied [10, 11].

Images were standardized by positioning the Frankfort horizontal plane parallel to the floor, and axial orientation was defined using the crista galli and the foramen magnum, ensuring correct anatomical alignment for the measurements. This standardization ensured measurement reproducibility and reliability [11].

**Fig. 1:**
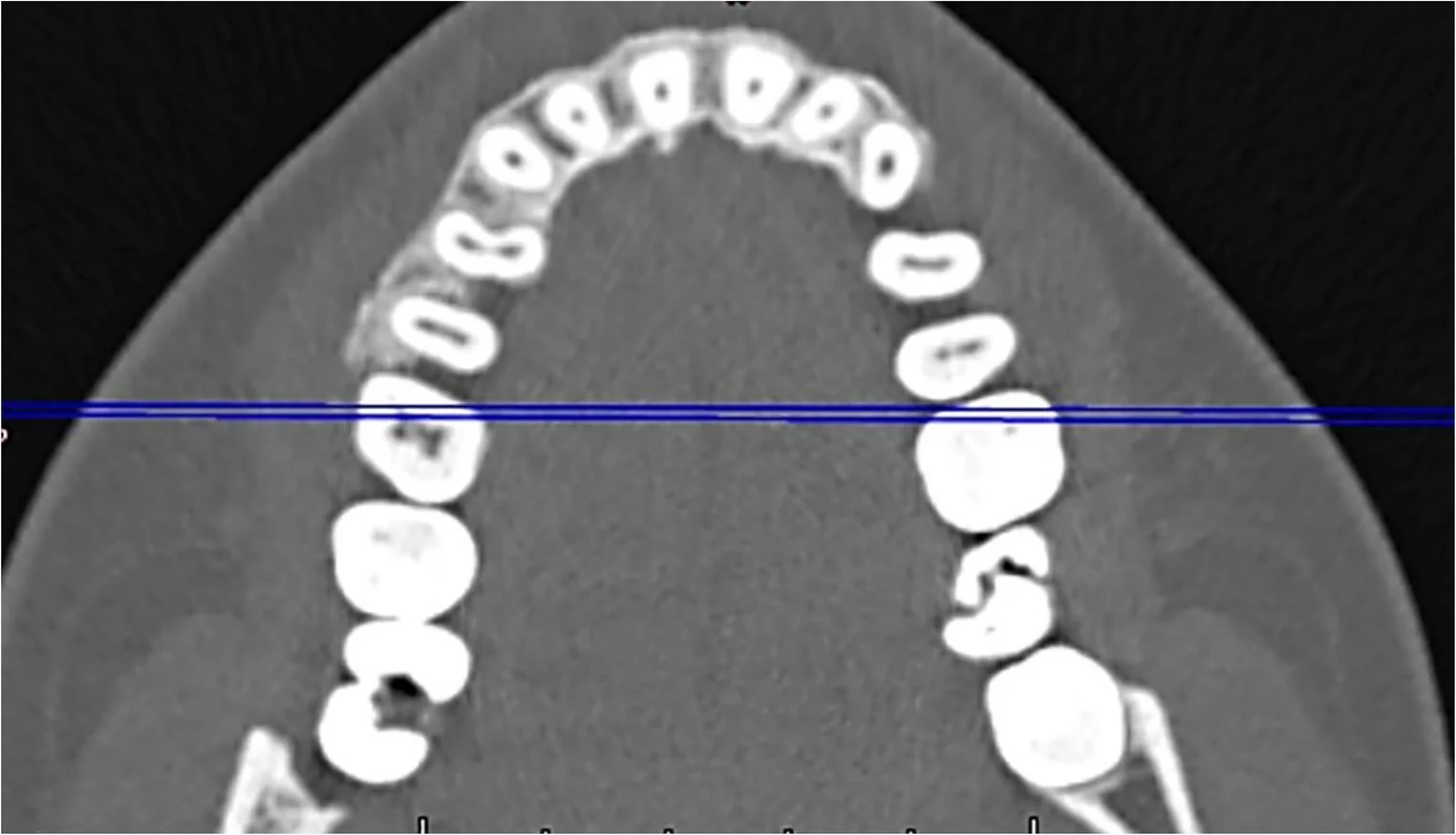
Axial tomographic slice located 5 mm anterior to the opening of the root canal of the permanent right maxillary first molar, used as the anatomical reference for defining the area of nasal septum analysis

Coronal CT slices were selected beginning at the level passing through the center of the mesiobuccal root canal of the permanent right maxillary first molar. Two additional frontal slices, one located 5 mm anterior and another 5 mm posterior to this reference point, were also analyzed and the mean of the measurements obtained from the three frontal slices was considered for statistical analysis. (Fig. 1).

**Fig. 2:**
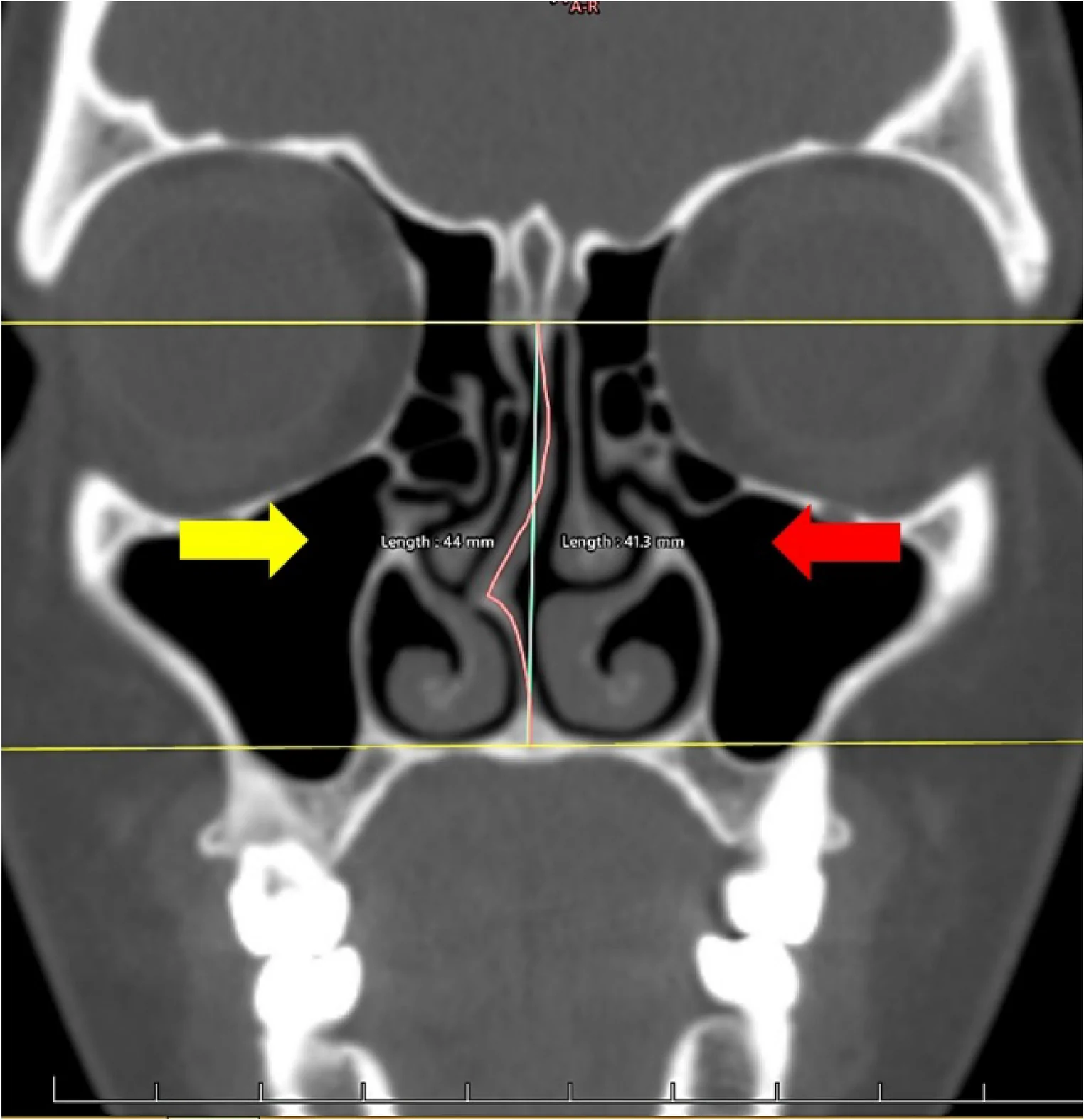
Measurements performed on a coronal computed tomography slice. Yellow horizontal lines indicate the anatomical reference planes used to delimit the boundaries of the nasal cavity; the blue vertical line represents nasal cavity height, and the pink curved line illustrates the nasal septum contour

The height of the nasal cavity was measured using a straight vertical line from the nasal floor to the roof of the cavity. The contour of the nasal septum was measured by the length of a curved line following the septal morphology (Fig. 2). The anatomical reference points used were a horizontal line passing through the nasal floor and the cribriform plate of the ethmoid bone, respectively.

The ratio between these measurements indicated the degree of nasal septal deviation (NSD), with values greater than 1 representing septal deviation. This methodology, validated by Aziz et al. [10], allows an objective and quantifiable assessment of nasal septal deviation.

### Examiner calibration

To ensure measurement reproducibility and reliability, inter-examiner calibration was performed. In accordance with methodological recommendations 20% of the sample was randomly selected for this procedure [12]. Two examiners (J.F.F. and C.R.C.F.) independently performed point identification and measurements at two different time points, with a two-week interval between assessments.

Agreement was evaluated using the intraclass correlation coefficient (ICC), calculated in IBM SPSS Statistics for Windows, version 25.0 (IBM Corp., Armonk, NY, USA). Reliability was interpreted according to the following criteria: excellent for values above 0.90; good for values between 0.75 and 0.90; moderate for values between 0.50 and 0.75; and poor for values below 0.50.

### Statistical analysis

Data were tabulated and statistically analysed using jamovi, version 2.6.11 (The jamovi project, Sydney, NSW, Australia). The distribution of the data was assessed with the Shapiro–Wilk test and the homogeneity of variances with Levene’s test. Intragroup comparisons between T0 and T1 were performed with the paired Student’s t test, and intergroup comparisons with the independent Student’s t test; the Wilcoxon signed-rank test and the Mann–Whitney U test were The level of significance was set at 5% (*p* < 0.05).

## Results

### Intraclass correlation coefficient

The Intraclass Correlation Coefficient (ICC) analysis was performed to assess the reproducibility of measurements related to nasal height and nasal septal deviation. For nasal height, the ICC was 0.969 based on the mean measurements between observers 1 and 2 at time point T0, and 0.942 at time point T1 (Table 1). Regarding the nasal septum contour, the ICC values were 0.974 at T0 and 0.938 at T1.

**Table 1:** Intraclass correlation coefficient (ICC) for nasal cavity height and septal contour measurements, assessed by observers 1 and 2 at T0 and T1, with a 15-day interval between evaluations

Variable	Time point	ICC (average measures)	Interpretation
Nasal cavity height	T0	0.969	Excellent
Nasal cavity height	T1	0.942	Excellent
Septal contour	T0	0.974	Excellent
Septal contour	T1	0.938	Excellent

*ICC* intraclass correlation coefficient

The obtained values demonstrate a high level of agreement between examiners and indicate that the measurements present good reproducibility. These findings confirm the adequacy of the calibration training performed by both examiners, supporting the reliability of the measurements for subsequent analyses.

### Statistical analysis

The final sample comprised 33 patients submitted to SARME, of whom 21 (63.64%) were treated with a Hyrax expander and 12 (36.36%) with a transpalatal distractor. The mean age was 24.6 ± 5.9 years in the Hyrax group and 33.2 ± 10.4 years in the transpalatal distractor group. Sex distribution was similar between the groups, and baseline clinical characteristics showed no differences relevant to the outcomes analysed.

Data distribution was assessed using the Shapiro–Wilk normality test, and homogeneity of variances was evaluated using Levene’s test. All analysed variables showed a normal distribution, and variances were homogeneous.

Intragroup analysis revealed no statistically significant changes for any of the variables studied (Table 2). In Group 1 (transpalatal distractor), mean nasal cavity height remained stable (45.40 ± 4.00 mm at T0 and 45.39 ± 4.23 mm at T1; *p* = 0.982). Septal contour measurements ranged from 46.40 ± 4.00 mm to 46.55 ± 4.22 mm (*p* = 0.790), and NSD values changed from 1.020 ± 0.015 to 1.030 ± 0.012 (*p* = 0.517).

In Group 2 (Hyrax), mean nasal cavity height was 45.10 ± 3.62 mm at T0 and 45.28 ± 3.52 mm at T1 (*p* = 0.588). Septal contour measurements averaged 46.80 ± 4.21 mm at T0 and 46.43 ± 3.89 mm at T1 (*p* = 0.250). NSD values showed a mean of 1.040 ± 0.032 at T0 and 1.020 ± 0.028 at T1 (*p* = 0.070).

No statistically significant differences were observed in the intergroup comparisons (Table 3). Mean nasal cavity height values were similar between the Hyrax group (45.28 ± 3.52 mm) and the transpalatal distractor group (45.39 ± 4.23 mm; *p* = 0.932). Septal contour measurements also did not differ significantly between groups (46.43 ± 3.89 mm and 46.55 ± 4.22 mm; *p* = 0.936), and NSD values were comparable between the Hyrax (1.020 ± 0.028) and transpalatal distractor (1.030 ± 0.012) groups (*p* = 0.901).

**Table 2:** Intragroup comparison (preoperative and postoperative) for the Hyrax and transpalatal distractor groups

Variable	Group	T0 (mean ± SD)	T1 (mean ± SD)	Mean difference (T1 − T0)	95% CI	*p* value	Test
Nasal cavity height (mm)	Hyrax	45.10 ± 3.62	45.28 ± 3.52	+ 0.14	−0.39 to + 0.67	0.588	a
	Transpalatal distractor	45.40 ± 4.00	45.39 ± 4.23	−0.01	−1.13 to + 1.11	0.982	a
Septal contour (mm)	Hyrax	46.80 ± 4.21	46.43 ± 3.89	−0.34	−0.94 to + 0.26	0.250	a
	Transpalatal distractor	46.40 ± 4.00	46.55 ± 4.22	+ 0.14	−1.00 to + 1.28	0.790	a
NSD (ratio)	Hyrax	1.040 ± 0.032	1.020 ± 0.028	−0.011	−0.023 to + 0.001	0.070	a
	Transpalatal distractor	1.020 ± 0.015	1.030 ± 0.012	+ 0.003	−0.008 to + 0.014	0.517	a

*NSD* nasal septal deviation, *SD* standard deviation, *CI* confidence interval. Linear variables are presented with two decimal places and the NSD ratio with three decimal places. A positive mean difference indicates an increase from T0 to T1.

a Independent Student’s t test, b Mann–Whitney U test

**Table 3:** Intergroup comparison between the Hyrax and transpalatal distractor groups at T1

Variable	Hyrax (mean ± SD)	Transpalatal distractor (mean ± SD)	Mean difference	95% CI	*p* value	Test
Nasal cavity height (mm)	45.28 ± 3.52	45.39 ± 4.23	−0.12	−2.91 to + 2.68	0.932	a
Septal contour (mm)	46.43 ± 3.89	46.55 ± 4.22	−0.12	−3.07 to + 2.84	0.936	a
NSD (ratio)	1.020 ± 0.028	1.030 ± 0.012	−0.001	−0.019 to + 0.016	0.901	b

*NSD* nasal septal deviation, *SD* standard deviation, *CI* confidence interval. A negative mean difference indicates a lower value in the Hyrax group.

a Independent Student’s t test, b Mann–Whitney U test

## Discussion

The aim of the present study was to evaluate the occurrence of changes in nasal septal position following Surgically Assisted Rapid Maxillary Expansion (SARME), comparing the use of tooth-borne and bone-borne expanders through tomographic analysis of nasal cavity height and nasal septum contour, the ratio of which determined the degree of septal deviation. The results demonstrated that the procedure did not promote statistically significant changes in either nasal height or nasal septal deviation, regardless of the type of expander used. Both in intragroup analyses and in comparisons between the groups treated with the Hyrax expander and the transpalatal distractor, the evaluated variables remained stable during the postoperative period, with minimal mean differences and confidence intervals that included the null value. These findings suggest that SARME, when performed according to the protocol adopted in this study, does not exert a significant impact on nasal septum morphology, nor does the type of expander influence this outcome.

Although the present results did not demonstrate significant changes in nasal septal position after SARME, Seidita et al., in an evaluation of 29 adult patients, reported mean changes in septal deviation ranging from 0.4 to 1.2 mm, more pronounced in the anterior region of the osseous septum. Despite these findings, the authors themselves emphasized that most of the observed changes were subclinical in nature, with only 6.8% of patients presenting clinically perceptible septal deviation associated with reduced nasal airflow [6].

However, a relevant methodological difference between the studies should be highlighted. The assessment performed by Seidita et al. was based on measuring the distance between the nasal septum and the lateral walls of the nasal cavity at different levels, a parameter that may reflect not only true septal displacement but also changes resulting from bone remodeling and lateral displacement of the nasal walls following maxillary expansion. Therefore, the small variations observed may represent global spatial modifications of the nasal cavity rather than a pathological septal deviation per se, as discussed by the authors themselves.

The surgical team responsible for the present study chose to perform an operative technique without pterygomaxillary suture separation and without nasal septum release. Multiple reviews comparing SARME performed with or without pterygomaxillary disjunction have demonstrated no statistically significant differences in the degree of transverse skeletal or dental gain. More recently, a systematic review with meta-analysis, showed that pterygomaxillary disjunction is not a determining factor for the success of maxillary expansion in adults, although it is associated with a more pronounced expansion pattern in the posterior palatal region, without consistent clinical advantages to justify its routine use [13].

This interpretation is reinforced by a recent multicentre study in which two minimally invasive bone-anchored expansion protocols were directly compared in 40 adults, differing precisely in the inclusion or omission of pterygomaxillary disjunction and nasal septum disinsertion [14]. Both protocols produced significant transverse gains, with no difference between them in the anterior (4.8 mm versus 5.7 mm; *p* = 0.44) or the posterior (2.9 mm versus 3.8 mm; *p* = 0.20) region, and neither the linear septal deviation from the midline nor the septal deviation angle changed significantly in either group (*p* = 0.099 and *p* = 0.062 in the group operated without the manoeuvres; *p* = 0.072 and *p* = 0.16 in the group in which they were performed). Mild septal displacement was identified in 35% of the sample, without statistical or clinical relevance, and the omission of septal release was not associated with an inferior expansion outcome. Obtained with a different measurement approach — linear and angular deviation from the midline, assessed within thirty days of the completion of expansion — and in a different surgical context, these findings converge with our own and reinforce the argument that neither pterygomaxillary disjunction nor nasal septum disinsertion is indispensable, either for achieving adequate expansion or for preserving the position of the nasal septum.

Similar findings were reported by Reinbacher et al., who evaluated 25 patients undergoing SARME without nasal septum release and without pterygomaxillary disjunction. Tomographic analysis of the nasal septum, combined with clinical and endoscopic assessment of respiratory function, did not reveal statistically significant changes, leading the authors to conclude that the observed deviations were minimal and predominantly subclinical [4]. Likewise, Jensen and Rodrigo-Domingo comparing patients treated with and without nasal septum release, found no differences in septal angular deviation, nasal appearance, or intranasal respiratory function in the postoperative period [15].

Dias et al. assessed the position of the nasal septum in 26 patients using linear measurements on tomographic examinations at four equidistant points along its inferior portion, comparing preoperative, immediate postoperative, and late postoperative periods after SARME performed without pterygomaxillary separation and without septal release. The authors did not identify lateral displacement or loss of symmetry between the right and left sides at any of the analyzed points, indicating nasal septum stability even in the absence of septal osteotomy [16].

Therefore, the present study contributes to the growing body of academic discussion suggesting that pterygomaxillary suture release is not essential to achieve satisfactory transverse correction, nor does routine nasal septum release result in measurable improvements in septal position. These findings support the adoption of less invasive surgical protocols and the individualization of treatment according to the severity of the maxillary deformity.

Beyond aspects related to the surgical technique, the most relevant point evaluated in the present study concerns the influence of the type of expander used and its impact on the nasal septum. The effects of maxillary expansion and SARME on the nasal cavity began to be investigated in the late 1980s and remain, to this day, an ongoing subject of research. However, to date, no studies have been identified that aimed to directly measure the impact of expander type on the nasal septum in this specific procedure [17–19].

The literature consistently demonstrates that, although both devices promote adequate expansion, bone-borne expanders tend to produce greater skeletal involvement, with reduced dental tipping and a more homogeneous distribution of forces throughout the nasomaxillary complex [20–21]. In contrast, tooth-borne expanders exhibit a greater dentoalveolar component and higher stress concentration in the alveolar processes [3, 22]. From a biomechanical standpoint, it was hypothesized that these differences could have distinct effects on structures adjacent to the maxilla, including the nasal cavity and the nasal septum.

Despite these biomechanical differences, neither expander type was associated with a significant change in nasal septum position in the present sample, the observed variations being numerically small and statistically non-significant. This result is consistent with the septal stability reported after expansion protocols using bone-borne and bone-anchored appliances [4, 14–16], and specifically with the findings of da Rosa et al., who observed comparable and non-significant septal displacement in two groups treated with bone-anchored expanders of different design, one prefabricated and one customised [14]. Taken together, these data suggest that the anchorage system and the design of the expander, although determinant for the distribution of the skeletal and dentoalveolar components of expansion [20–22], are not determinant for the postoperative position of the nasal septum in adults. Although that study was not designed to isolate the effect of device type on the septum, its results are consistent with the present finding that appliance design does not determine septal behaviour.

It is noteworthy that the effects of maxillary expansion on septal morphology are more frequently reported among children and adolescents undergoing orthopaedic expansion. In these populations, a reduction in the degree of septal deviation has been observed after rapid maxillary expansion, attributed to the release of the septal attachment to the maxillary crest at a stage in which the septum is still growing and capable of remodelling [10, 11, 23]. It is well established, however, that nasal septum growth progressively ceases between 16 and 18 years of age [23]. This maturational factor may explain the absence of significant changes observed in the present adult sample, in which greater structural rigidity is expected in response to expansion forces, regardless of the type of anchorage used.

The magnitude of the observed changes deserves consideration. In both groups, the mean changes in nasal cavity height and septal contour did not exceed 0.35 mm, and the mean change in the NSD ratio did not exceed 0.011. These magnitudes are of the same order as, or smaller than, the spatial resolution of the acquisition protocol employed (voxel size 0.38 × 0.38 × 0.43 mm; slice thickness 0.5 mm), and should therefore be interpreted as lying within the range of measurement error rather than as a detectable anatomical change. They are also smaller than the displacement of 0.4 to 1.2 mm described by Seidita et al. as expected after SARME [6] and than the mean increase of 0.3 to 0.4 mm in linear septal deviation reported by da Rosa et al. [14]. The mean NSD ratio remained between 1.020 and 1.040 at both time points and in both groups, within the range interpreted as an absence of relevant deviation by the method adopted [10].

Two observations of clinical interest follow, within the limits of what the present design allows. Since septal position remained stable under a protocol that deliberately omitted both pterygomaxillary disjunction and septal release, the routine performance of septal disinsertion does not appear to be justified by a concern for septal position alone, and avoiding this manoeuvre spares the patient the risk of intraoperative and postoperative bleeding and of nasal mucosal retraction associated with it [4, 14–16]. In addition, since the type of expander did not influence septal behaviour, the choice of device may be guided by other clinical criteria — palatal anatomy and bone availability, the expected ratio between skeletal and dentoalveolar expansion, the magnitude of expansion required, patient comfort and cost.

Some limitations of this study should be considered. The sample size and the postoperative follow-up period may limit the detection of subtle or late changes in septal morphology. In addition, the analysis was based exclusively on retrospective data obtained from tomographic examinations. No a priori sample size calculation was performed, as the sample was determined by the number of eligible cases available in the study period, and the two groups were of unequal size; the study was therefore not designed to detect small differences between devices. The absence of functional outcome measures — rhinomanometry, acoustic rhinometry, endoscopic assessment or validated symptom questionnaires — also prevents any direct inference about nasal breathing, and the six-month follow-up does not allow late remodelling of the septum to be assessed. Future studies with larger samples, longer follow-up periods and the integration of three-dimensional analyses with functional respiratory assessments may contribute to a more comprehensive understanding of the impact of SARME and expander type on nasal structures.

## Conclusion

SARME did not promote statistically significant changes in nasal septal deviation or in nasal cavity height. The type of expander used (tooth-borne or bone-borne) did not influence the evaluated outcomes, and the observed modifications were minimal and of a magnitude below the threshold of clinical perceptibility. From a clinical perspective, despite the limitations inherent to the study design, the results indicate that the choice of surgical technique and expander type should be based on individual patient assessment, as no significant differences were observed regarding their impact on the nasal septum.

## Data Availability

All data supporting the findings of this study are available within the paper.
